# Mapping Characteristics, Applications, and Implementation Challenges of Virtual Communities in Cancer Care: NASSS Framework-Informed Scoping Review

**DOI:** 10.2196/73093

**Published:** 2025-10-22

**Authors:** Jiapei Dong, Yi He, Lili Tang

**Affiliations:** 1 Key Laboratory of Carcinogenesis and Translational Research (Ministry of Education/Beijing), Department of Psycho-Oncology, Peking University Cancer Hospital & Institute, 52 Fucheng Road, Haidian District, Beijing, 100142, China, 86 10 88196648; 2 Inner Mongolia Cancer Center, Peking University Cancer Hospital (Inner Mongolia Campus)/Affiliated Cancer Hospital of Inner Mongolia Medical University, Hohhot, China

**Keywords:** cancer care, digital health interventions, emotional well-being, health disparities, implementation challenges, NASSS framework, oncology, patient outcomes, PRISMA, psychosocial support, user engagement, virtual community

## Abstract

**Background:**

Patients with cancer frequently experience psychological and social challenges, including depression, anxiety, and isolation, which are often intensified by treatment side effects and unmet psychosocial needs. Conventional support systems are often inaccessible, under-resourced, or poorly tailored to diverse patient populations. In this context, virtual communities have emerged as promising alternatives that enable peer interaction, emotional support, and information exchange. However, their implementation and sustainability are influenced by complex sociotechnical and organizational factors that remain underexplored.

**Objective:**

This scoping review applies the Non-adoption, Abandonment, Scale-up, Spread, and Sustainability (NASSS) framework to examine how virtual communities have been implemented in cancer care. It aims to identify key barriers and facilitators, evaluate the alignment between platform features and user needs, and synthesize evidence to inform sustainable integration into care systems.

**Methods:**

A systematic search was conducted across 6 databases (PubMed, Scopus, Embase, Web of Science, PsycINFO, and CINAHL), covering studies published between 2019 and 2024. Eligible studies were empirical and reported on the development, implementation, or evaluation of virtual communities for patients with cancer. Data were extracted using a structured Non-adoption, Abandonment, Scale-up, Spread, and Sustainability–based matrix and synthesized thematically across diverse research designs.

**Results:**

The search yielded 322 records, of which 175 full-text studies were assessed for eligibility, and 25 studies were included in the review. These studies covered a range of virtual community formats used by patients with cancer. All included studies reported psychosocial benefits, including reduced loneliness, improved emotional well-being, and greater opportunities for experience sharing. However, key challenges remained, such as low user retention, limited participation from underrepresented groups, and difficulties integrating these platforms into existing health care systems. Few studies reported longitudinal follow-up or detailed engagement metrics, limiting insights into long-term effectiveness.

**Conclusions:**

Virtual communities show strong potential to address the psychosocial needs of patients with cancer, especially in underserved populations. However, to ensure long-term effectiveness, attention must be paid to inclusivity, user retention, ethical considerations, and system-level integration. Future research should incorporate standardized metrics, longitudinal designs, and equity-oriented approaches to optimize the development and implementation of virtual communities in cancer care.

## Introduction

Advancements in medical technology, early diagnostic practices, and innovative treatment modalities have significantly improved cancer survival rates [1]. However, many patients with cancer, particularly those in long-term survivorship, often face a heightened risk of mental health issues, including depression and anxiety, with incidence rates up to 4 times higher than those in the general population [2]. Additionally, the toxic side effects of anticancer drugs, such as pain, fatigue, and nausea, frequently lead to profound psychological, social, and spiritual distress [3,4]. These intertwined psychosocial challenges undermine patients’ social adaptability and self-efficacy, exacerbating psychological and social isolation [5,6]. These unmet needs substantially reduce quality of life and are associated with increased mortality risks [7].

Despite this critical need, existing mental health support systems remain inadequate, with only 10% of patients with cancer receiving the formal psychosocial support they may require [8]. Access barriers and resource disparities disproportionately affect underserved populations, such as those with lower education levels, financial constraints, or inadequate social support networks [9-11]. These disparities highlight an urgent need for accessible and scalable solutions to address the psychological and social needs of patients with cancer.

The rapid evolution of digital technologies has positioned virtual communities as valuable platforms for patients with cancer seeking psychosocial support. These online networks transcend geographical and temporal barriers, enabling individuals to exchange information, share experiences, and offer emotional support [12]. Virtual communities empower patients, foster psychological resilience, and alleviate feelings of loneliness [13,14], providing a flexible and accessible alternative to traditional support systems. Anonymity further encourages open expression, allowing patients to share sensitive experiences and seek guidance in a safe environment [12].

Despite their potential, virtual communities face substantial challenges in implementation and sustainability [15]. Key barriers include insufficient organizational resources, limited system interoperability, and gaps in policy frameworks and privacy protections, all of which undermine patient trust and participation [16-19]. Moreover, linear evaluation models commonly used in digital health research fail to capture the dynamic complexities of virtual communities, including technological evolution, user behavior patterns, and multistakeholder collaboration [20,21]. These implementation challenges call for a more comprehensive framework to guide the design, implementation, and optimization of virtual communities in cancer care.

The Non-adoption, Abandonment, Scale-up, Spread, and Sustainability (NASSS) framework provides a structured methodology to analyze the adoption and implementation of digital health technologies [22]. By addressing key dimensions such as condition characteristics, technology design, value proposition, user attributes, organizational capacity, external context, and dynamic interactions, the NASSS framework supports a comprehensive analysis of the opportunities and challenges associated with virtual communities in cancer care [22].

This scoping review is the first to apply the NASSS framework to virtual communities in oncology. It aims to map how existing literature has examined their implementation, sustainability, and alignment with patient and system-level needs. The review highlights the concept of feature alignment, which refers to how well platform functions correspond with user expectations, clinical roles, and infrastructure demands.

From an implementation science perspective, the review examines contextual factors such as stakeholder involvement, organizational capacity, and system interoperability that shape the real-world adoption, integration, and long-term sustainability of virtual communities in cancer care [23]. Through this synthesis, it identifies key research gaps and emerging patterns to guide future development and integration of virtual communities into oncology care.

## Methods

### Study Design and Analytical Framework

This study was designed as a scoping review to explore how current literature addresses the implementation of virtual communities in cancer care. Given the diversity and complexity of existing studies, a scoping review was chosen as the most appropriate approach to systematically map available evidence, identify structural features, and highlight research gaps in the implementation of virtual communities in cancer care. We followed the 5-stage methodological framework proposed by Arksey and O’Malley [24] and refined by Levac et al [25], including: (1) identifying the research question; (2) identifying relevant studies; (3) selecting studies; (4) charting the data; and (5) collating, summarizing, and reporting the results. The PRISMA-ScR (Preferred Reporting Items for Systematic Reviews and Meta-Analyses extension for Scoping Reviews) checklist was used to guide reporting (see Checklist 1 ) [26].

To organize data and guide synthesis, we used the NASSS framework. This framework provides 7 domains for analyzing the implementation of digital health interventions. In this study, it was used not for outcome evaluation but as a conceptual structure for identifying and categorizing descriptive content relevant to the implementation of virtual communities.

### Definition of Virtual Community

A “virtual community” in this review refers to any digital environment, including online forums, social media groups, web-based discussion spaces, or mobile apps, that facilitates peer interaction, emotional exchange, informational support, or psychosocial connection among individuals affected by cancer. Platforms were included regardless of whether they were moderated, open or closed, professionally integrated, or patient-led.

For clarity, the term “virtual community” is used throughout this review as the overarching concept. Terms such as “platform” or “online support group” appear only when describing specific formats or technical implementations. The term “patients with cancer” refers to individuals at any phase of cancer care, including diagnosis, active treatment, and survivorship. “Cancer survivors” is used only when included studies specifically focus on post-treatment experiences. This distinction ensures terminological consistency and conceptual coherence.

To be included, studies had to report content relating to the actual use or implementation of such virtual communities. This included user experiences, stakeholder opinions, usability or accessibility issues, organizational challenges, privacy concerns, or other aspects relevant to the NASSS domains.

#### Stage 1: Identifying the Research Question

The central research question was as follows: How do empirical studies address implementation-relevant features of virtual communities in cancer care, particularly in terms of technological design, user experience, organizational capacity, and stakeholder involvement, as outlined in the NASSS framework?

This question guided a broad but structured exploration of implementation-related features across diverse study types. To support this, clear eligibility criteria were defined to guide study selection, ensuring thematic relevance to the research question and alignment with the NASSS framework. The criteria are summarized in Textbox 1.

Textbox 1. **Eligibility criteria.**

**Inclusion criteria**

- Population: involved patients with cancer at any stage (diagnosis, treatment, survivorship, or recovery)
- Platform: focused on a virtual community as defined above
- Content: reported any implementation-relevant experiences or perspectives (eg, user feedback, stakeholder involvement, privacy concerns, platform accessibility, or organizational capacity)
- Scope: contained content mappable to at least 1 Non-adoption, Abandonment, Scale-up, Spread, and Sustainability domain
- Design: presented original empirical data (quantitative, qualitative, or mixed methods)
- Language and publication date: published in English between 2019 and 2024

**Exclusion criteria**

- Studies not involving patients with cancer
- Interventions that relied solely on telephone or SMS-based communication
- Reviews, commentaries, opinion papers, protocols, and conference abstracts
- Studies that mentioned virtual communities without describing any real-world usage, implementation experience, or system-level insight

#### Stage 2: Identifying Relevant Studies

A comprehensive literature search was conducted across 6 electronic databases: PubMed, EMBASE, SCOPUS, Web of Science (Core Collection), EBSCOhost, and PsycINFO. The search strategy was structured around 3 conceptual domains: virtual community, cancer, and psychosocial support. It combined both free-text keywords and Medical Subject Headings (MeSH) where applicable.

The final database search was conducted in September 2024, and the inclusion criteria were restricted to studies published between January 2019 and September 2024. In addition to database searches, we manually screened the reference lists of relevant reviews to identify potentially eligible studies. The full search strategy is presented in Multimedia Appendix 1.

Search terms were developed by the authors through internal discussion, based on preliminary literature screening and the objectives of this review. Keyword selection was informed by commonly used terminology found in prior empirical and review studies concerning cancer care, psychosocial support, and digital health. The terms were adapted to the syntax and indexing systems of each database, using field-specific tags such as “tiab” in PubMed, “SU” in PsycINFO, and “TS” in Web of Science.

Although our search retrieved review studies, these were excluded from the final synthesis and examined only to inform the conceptual orientation and background of this study.

#### Stage 3: Study Selection

All retrieved records were imported into *EndNote X* (version 9.3.3; Clarivate) for reference management. Duplicates were removed using both automated and manual procedures. Title and abstract screening was conducted independently by reviewers (JPD and YH) using the predefined eligibility criteria summarized in Textbox 1. Discrepancies between reviewers were resolved through discussion until consensus was reached.

Full-text studies were retrieved that appeared eligible or where inclusion could not be determined from the abstract. Full-text screening followed the same 2-level review procedure. Studies were excluded at this stage if they (1) lacked content related to the implementation or use of virtual communities, (2) focused solely on conceptual discussions or frameworks without empirical data, (3) involved populations not specific to cancer, or (4) did not meet the operational definition of virtual communities as specified in this review.

All inclusion and exclusion decisions were based exclusively on thematic relevance to the research question, rather than on study quality or methodological rigor. Studies were eligible regardless of whether they employed qualitative, quantitative, or mixed methods, provided they contained content relevant to the implementation of virtual communities in cancer care. This approach aligns with established scoping review methodology, which emphasizes conceptual alignment over quality-based filtering during study selection.

The results of the selection process, including the number of records retrieved, screened, excluded, and included, are reported in the *Results* section . A complete list of excluded studies and categorized exclusion reasons is provided in Multimedia Appendix 2 to ensure transparency.

#### Stage 4: Charting the Data

A structured data extraction process was conducted using a predefined Excel matrix (Multimedia Appendix 3) developed to ensure consistency and alignment with the NASSS framework. The extraction form included a set of general study characteristics (eg, publication year, country, cancer type, platform type) and 7 sets of domain-specific fields corresponding to the NASSS framework.

For each included study, we identified and recorded implementation-relevant content related to the following domains: (1) the condition, (2) the technology, (3) the value proposition, (4) the adopters, (5) the organization, (6) the wider system, and (7) embedding and adaptation over time. Each domain was operationalized through a set of indicators, and examples were provided to guide consistent application across studies. A comprehensive description of this operationalization process is provided in Multimedia Appendix 4, which outlines the conceptual definitions of each domain, illustrates how they are reflected in the literature, and details the specific data extraction fields used. The appendix also includes structured instructions for completing the extraction matrix and guidance on handling missing, inapplicable, or supplemental data, thereby enhancing the transparency and reproducibility of the review process.

The data extraction methodology was adapted from Miao et al (2022) with modifications to fit the focus and scope of this review [27]. All included studies were reviewed in full text, and relevant content was manually extracted. Transcription and minor translation were conducted when necessary to preserve clarity. Initial extraction was performed by JPD and independently verified by YH to ensure reliability and consistency.

To further capture the diversity of digital features and peer interaction models, supplementary data were extracted based on the Digital Health Intervention Taxonomy [28], including delivery formats, communication flow, and potential psychosocial benefits of user interaction.

#### Stage 5: Collating, Summarizing, and Reporting the Results

We conducted a narrative synthesis of the charted data. Guided by the NASSS framework, the results were thematically organized across its 7 domains [22]. Within each domain, we summarized implementation-related experiences, commonly reported challenges, and facilitating factors described in the included studies.

Instead of comparing intervention outcomes or evaluating effect sizes, the synthesis focused on identifying structural and experiential features of virtual communities that may influence their adoption, engagement, and long-term sustainability. This organization enabled us to examine how specific features aligned with user needs, organizational resources, and broader contextual factors.

Although considerable heterogeneity existed in study designs, participant characteristics, and technological formats, no formal comparative analysis was conducted. This approach aligns with the purpose of a scoping review, which emphasizes mapping the evidence rather than evaluating its effectiveness.

### Quality Assessment

The methodological characteristics of the included studies were descriptively assessed using the Mixed Methods Appraisal Tool (MMAT) [29]. The MMAT was applied to profile methodological strengths and limitations across qualitative, quantitative, and mixed methods studies. No studies were excluded based on MMAT scores, aligning with the scoping review’s focus on mapping evidence rather than evaluating quality. This descriptive profiling supports the interpretation of findings while maintaining transparency and reproducibility (see Multimedia Appendix 5).

## Results

### Study Selection

**Figure 1.**
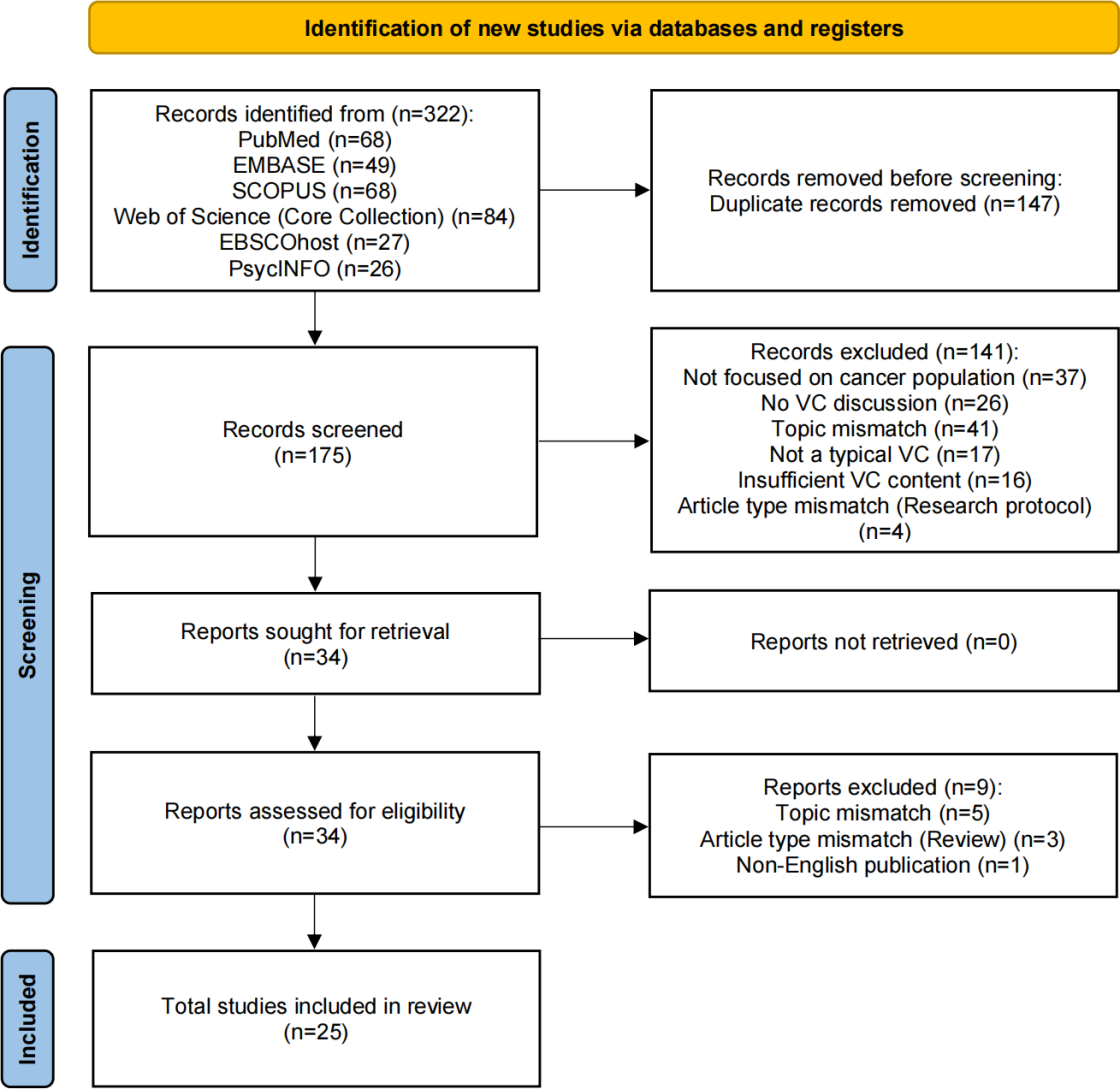
PRISMA-ScR (Preferred Reporting Items for Systematic Reviews and Meta-Analyses extension for Scoping Reviews) flow diagram detailing the study selection process. VC: virtual community.

The initial database search yielded 322 records. After removing 147 duplicates, 175 unique records remained for title and abstract screening. Based on relevance to the review objective and predefined eligibility criteria, 34 studies were selected for full-text review. Of these, 9 were excluded. Excluded studies included (1) studies not focused on populations with cancer; (2) studies not addressing the use or implementation of virtual communities; (3) publications ineligible for inclusion, such as reviews or research protocols; and (4) interventions using communication modes outside the defined scope, for example, telephone or SMS-only. These categories are summarized in the *Study Selection* section and listed in detail in Multimedia Appendix 2. The full study selection process, including the number of records screened and excluded at each stage, is illustrated in Figure 1 in the PRISMA-ScR flow diagram.

### Research Distribution and Trends

**Figure 2.**
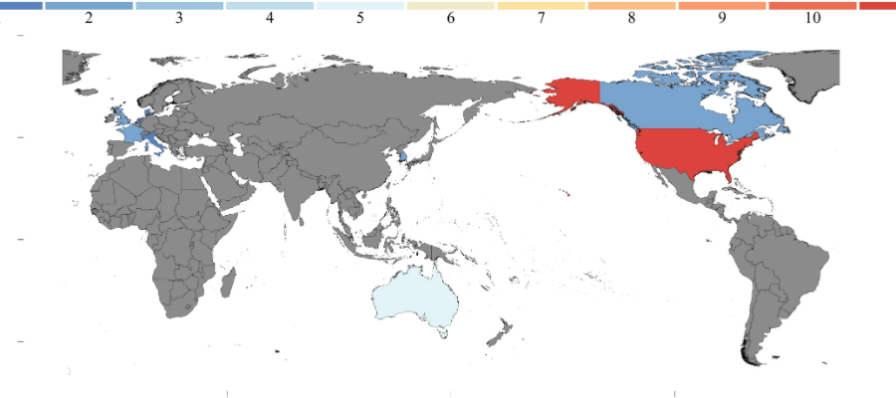
Geographic distribution of the included studies, illustrating the global landscape of virtual community implementation in cancer care (2019-2024). Color intensity indicates the number of studies per country, with darker shades representing higher publication volume.

This review includes 25 studies published between 2019 and 2024, from 9 countries (see Table 1 for publication year distribution). A total of 4 studies involved international collaborations, all of which included the United States. Research activity peaked in 2020, with 7 studies published that year, likely driven by the COVID-19 pandemic, which led to increased interest in virtual communities for health care delivery. A total of 5 studies specifically addressed the impact of COVID-19 [30-34]. Geographically, North America accounted for the majority of studies (n=14), followed by Europe (n=6), Australia (n=5), and Asia (n=2), with detailed regional distributions visualized in Figure 2.

**Table 1.** Distribution of included studies by year of publication, showing the temporal trend of research on virtual communities in cancer care (2019‐2024).

Publication year	Value, n (%)
2019	2 (8)
2020	7 (28)
2021	6 (24)
2022	4 (16)
2023	5 (20)
2024	1 (4)

### Research Design and Methods

Qualitative studies dominate the field, accounting for 52% (13/25) of the included studies, followed by mixed methods (5/25, 20%) and quantitative studies (4/25, 16%). Thematic analysis is the most commonly used approach (14/25, 56%), often in combination with semistructured interviews (7/25, 28%) or qualitative interviews (3/25, 12%). Quantitative studies primarily rely on survey-based designs (6/25, 24%), particularly in psychological research, using tools such as the Hospital Anxiety and Depression Scale to assess mental states. Emerging methodologies include open coding, content analysis, netnography, and text mining, with data sourced from public social media posts to purpose-built intervention platforms.

### Quality Assessment of Included Studies

The methodological quality of the included studies, assessed using the MMAT, varied across study types (see Multimedia Appendix 5). Most qualitative studies demonstrated clarity in research questions and analytical approaches, with limitations including non-representative sampling, limited reflexivity, and variable transparency in data interpretation. Quantitative studies generally met basic MMAT criteria but often relied on self-reported data from voluntary online participants, potentially introducing selection or response bias. Despite these limitations, all studies provided insights into user experiences, stakeholder engagement, and contextual factors relevant to virtual community implementation in cancer care.

### The NASSS Framework

#### Domain 1: Condition

Among single cancer types, breast cancer was most commonly examined, appearing in 7 studies (28%) [30,35-40]. Pan-cancer studies, which included mixed cancer types, were the largest category overall (11/25, 44%) [31,34,41-48]. Other cancers were less represented, including colorectal (1 study, 4% [49]), lung (1 study, 4% [33]), thyroid (1 study, 4% [50]), and prostate cancer (1 study, 4% [32]). At least 3 additional studies focused on rarer cancers such as brain tumors, soft tissue sarcomas, or acute myeloid leukemia (3/25, 12%) [51-53].

Studies addressed all stages of cancer, from early diagnosis to advanced disease. Breast cancer research encompassed stages 0-III and advanced disease [30,40], whereas lung cancer studies primarily focused on stage IV [33]. Thyroid cancer studies involved preoperative patients with comorbid anxiety and depression [50], and colorectal cancer studies examined later stages after treatment [49]. Some studies focused on recurrence or postoperative management [34,37].

Regarding age distribution, most studies (6/25, 24%) targeted patients aged 30‐49 years [30,32,35,37,38,50]. Older adults were underrepresented. Research also included specific populations such as adolescent and young adult (AYA) patients [31] and disabled survivors [35]. Gender participation was skewed toward females, particularly in breast cancer communities, with women constituting 66.7%‐82% of participants [30,36-39,46]. Participants were generally highly educated, middle-income, and urban; in contrast, rural or remote populations were included in only 3 studies (3/25, 12%) [31,38,50]. Psychological comorbidities such as anxiety and depression were occasionally reported, especially in thyroid cancer studies [50].

#### Domain 2: Technology

##### Current Applications of Technology in Virtual Communities

Virtual communities provide multiple engagement channels for patients with cancer and their support networks. These include interactive panels or in-app interaction boards (4 studies; 30,31,34,35); online health communities or forums (11 studies; 37-43,47,48,51,54); and social media platforms such as Facebook, Twitter, Instagram, and Reddit (8 studies; 33,36,44,46,49,50,52,53). In addition, custom apps (eg, WeCanManage, WalkON, Kræftværket, iaya) incorporate features such as private messaging and personalization [30,31,34,35]. Cross-platform availability and mobile-friendly design further increase usability [30,34,51,53].

At least 10 of 25 studies (40%) reported reliance on mainstream social media platforms, which offered both public and private spaces [40,44-46]. These platforms integrate private and public spaces to balance trust and openness, and hybrid models combine the benefits of peer intimacy with the scalability of open networks [45].

Technological integration across formats facilitates a more cohesive support experience. For example, responses in Q&A boards often reflect themes raised in discussion forums, and live interactions can later be archived for reflective or educational use [36,45].

##### Technological Features

###### Overview

Virtual community platforms are characterized by user-friendly interfaces, diverse functionality, and cross-platform compatibility. These aspects apply to both customized apps (eg, WeCanManage, WalkON, Kræftværket) and broader health communities (eg, Mayo Clinic Connect, breastcancer.org, Kanker.nl) [40,48].

###### User-Friendly Interfaces

Intuitive and simple interfaces enhance accessibility for patients with varying levels of technical skills. Platforms such as Kanker.nl and breastcancer.org were consistently described as easy to navigate [40,48], whereas the Cancer Survivor Network (CSN) and Cancer Council Online Community (CCOC) were noted for design features that complicated use among older participants [36,43]. By contrast, Mayo Clinic Connect was highlighted as a platform with an optimized design that supports patient engagement [38].

###### Diverse Functionality

Platforms vary in how they integrate communication and support features. Disease-specific forums, such as Thyroid Family [50] and Les Impatientes [39], focused narrowly on certain cancer types, whereas Kanker.nl combined multiple functions, including peer support, emotional support, and information resources [48].

###### Cross-Platform Compatibility

Accessibility across devices was another distinguishing factor. Mayo Clinic Connect and Kanker.nl supported both web and mobile use [38,48], enabling more flexible access, whereas the CCOC was limited to web-only participation [47]. Social media platforms such as Facebook and Reddit enhance accessibility by facilitating seamless device transitions.

### Anonymization Design

Anonymization is reported in 9/25 (36%) studies as a common strategy in virtual communities to protect privacy and encourage open sharing, though its effects on engagement vary. Social media platforms such as Reddit adopt pseudonymity, enabling candid discussions on sensitive topics [42,46,52]. Twitter and Instagram build community through hashtags and visual content [44,53], while Facebook groups like COLONTOWN balance pseudonymity with real-name credibility [33,49]. Customized apps demonstrate different models: WalkON emphasizes openness without anonymization [30], Kræftværket permits anonymity at the expense of deeper ties [31], and iaya allows users to choose between anonymous and real-name identities [34]. Similarly, health-specific platforms like CCOC, Thyroid Family, and Les Impatientes provide anonymity to create safe spaces for emotional support [39,41,47,50,54]. At least 3 (12%) studies noted that anonymity can hinder trust, sustained conversations, or the formation of deeper relationships [31,39,45].

### Intervention Design

Customized apps like WalkON and Kræftværket provide tailored interventions, including health monitoring, educational modules, and automated data collection [30,31], offering support beyond basic peer interaction. However, only 4/25 (16%) studies evaluated such apps, and integration with clinical workflows or personalized care plans was reported in just 2/25 (8%) studies (Multimedia Appendix 3). Several studies (5/25, 20%) still relied on traditional data collection methods, such as paper or static questionnaires [30,31,38,41,50]. Mobile apps with feedback mechanisms and behavioral data analytics, as in iaya, show potential to enhance personalization while protecting privacy [30,31,34].

### Privacy and Data Management

Ensuring privacy and data security is critical for patient trust. Platforms like Kræftværket (Denmark) comply with General Data Protection Regulation (GDPR) [31], and iaya (USA) follows Health Insurance Portability and Accountability Act regulations with encrypted data transmission and storage [34]. However, the majority of studies (23/25, 92%) did not provide detailed descriptions of privacy policy implementation or assess whether users fully understood how their data were handled.

### Domain 3: Value Proposition

Virtual communities play an important role in the psychosocial rehabilitation of patients with cancer, addressing needs in informational support, emotional comfort, psychological empowerment, and social interaction. Platforms such as Mayo Clinic Connect, CSN, and Reddit provide structured information, peer reassurance, and emotional support, which can help reduce feelings of loneliness and anxiety, particularly during diagnosis and treatment [36-40]. For AYA patients, platforms such as Kræftværket have been reported to reduce isolation, strengthen coping abilities, and provide additional support for marginalized subgroups, including women experiencing reproductive or identity-related concerns during survivorship [31,34,46]. Tailored designs support diverse patient needs: prostate cancer survivors use gender-sensitive spaces for lifestyle and symptom management [32], patients with rare cancers like soft tissue sarcoma exchange clinical information and personal experiences [51], patients with lung cancer engage in private groups for individualized guidance [33], and patients with brain cancer prefer anonymous settings emphasizing emotional safety [52]. Mobile apps like WeCanManage integrate mindfulness exercises with peer interaction features, enhancing symptom management and quality of life [35].

On the supply side, virtual communities contribute to health care system efficiency by reducing resource burden and operational costs [30]. At least 2 studies mentioned that some platforms rely on low-cost designs, volunteer moderation, or mobile-based infrastructures to maintain accessibility and scalability [39,49]. Communities also facilitate patient engagement in decision-making and communication, improving provider-patient relationships [33,38,45], and support efficient data collection through surveys, health monitoring tools, and personalized support [42,50].

### Domain 4: Adopters

#### Patient Needs and Expectations

Patients in virtual communities assume diverse roles, including newcomers seeking information, altruists offering emotional support, and autonomous users responding to others’ inquiries [47,49]. These roles influence engagement and participation patterns. Patients’ needs change as their illness progresses. Patients with early-stage cancer generally prioritize authoritative information, whereas patients with advanced-stage cancer focus on psychological support for coping with disease progression and existential anxiety [46,50]. Patients with rare cancers often experience social isolation and require tailored services to address specific psychosocial and informational needs [45].

Demographic factors such as gender and age also shape participation. Women are more likely to seek emotional support online, whereas men may prefer offline connections or avoid disclosure, particularly in conditions like prostate cancer, where side effects may be perceived as embarrassing [31,32]. Younger, tech-savvy patients often prefer interactive platforms such as WhatsApp and Instagram, while older patients tend to use simpler, health care provider–endorsed resources [38,45].

Some patients eventually disengage from virtual communities, either because platforms do not meet their deeper emotional needs or because they wish to distance themselves from a persistent “sick role” identity [31,33,37,43,45]. Younger patients often prefer interactive platforms such as Instagram, while older patients gravitate toward more information-focused platforms like Facebook [38,43,47,53].

#### User Experience and Satisfaction

Interface design plays a pivotal role in user satisfaction and engagement. Patients generally prefer clear page layouts and straightforward navigation, which allow them to access information quickly and with minimal effort [34]. In contrast, design flaws such as small fonts, ambiguous icons, and overly layered menus can diminish usability, particularly for older adults or those with limited technical literacy [35,54]. Adolescents and younger users tend to favor interactive and visually engaging interfaces that foster a sense of belonging and encourage participation [31].

Functionality is also central to patient experience. Patients in rural or under-resourced areas often face challenges due to limited access to devices or insufficient digital skills. Some advanced features, such as community filters or resource search tools, may unintentionally create barriers if not well designed [32,54].

Personalized support further shapes users’ perception of community value. Patients appreciate the ability to connect with others who share similar diagnoses, treatment paths, or life contexts [31,34]. Closed groups using real names and profile images often foster deeper engagement, whereas open communities may become overly generalized or fragmented [49]. Demographic preferences also influence user needs: younger patients typically seek real-time interaction and peer feedback, while older individuals prioritize reliable information and ease of use [31,38]. Patients with rare diseases often value expert-informed guidance [34,45].

The quality of interpersonal interaction is another determinant of user satisfaction. Supportive peer communication can alleviate loneliness, validate experiences, and provide practical advice [34]. Conversely, low engagement, such as unresponsive posts, may undermine community value [47]. Negative or distressing content may amplify anxiety and reduce optimism, underscoring the importance of safeguards within community environments [34,44,45].

#### Role of Health Care Providers

Health care providers contribute to virtual communities primarily by offering evidence-based medical information, addressing misconceptions, and supporting symptom management and counseling. Studies show that clinician involvement through moderation, direct interaction, or content provision enhances user trust and engagement, as patients are more likely to rely on information verified or delivered by qualified professionals [36,45,51].

Beyond providing information, health care professionals facilitate meaningful interactions, offer emotional reassurance, and validate patient experiences within virtual communities. While anonymity can promote openness and reduce stigma, it may also reduce trust and the quality of interpersonal engagement [45,50]. There is only indirect evidence that the presence of health care providers may stabilize these dynamics, supporting both emotional expression and informational integrity (see Multimedia Appendix 3).

Clinicians are reported to participate in various activities, including community moderation, hosting live Q&A sessions, and co-designing educational content tailored to patient needs [34,42,45,50]. Their involvement contributes to more timely and individualized responses to patient concerns, enhancing the perceived credibility and legitimacy of the community [36,45,51].

#### Role of Technology Developers

Technology developers play a key role in creating virtual communities that are both usable and clinically relevant. Their work extends beyond technical implementation to shaping how patients access, interpret, and interact with health-related information (see Multimedia Appendix 3 for details) [34,35]. A user-centered design approach is particularly important for patients with cognitive or functional impairments. Adjustments such as font scaling, simplified navigation, and audio or text customization can reduce technical barriers and improve accessibility [35].

Collaboration among developers, clinicians, and patients contributes to both technical usability and the integration of clinical validity, emotional resonance, and cultural relevance within digital platforms [34]. Developers also embed safeguards, including identity verification, content filters, and data protection measures to ensure compliance with regulations and maintain user trust, especially among older adults [42,45].

### Domain 5: Organization (Internal Factors)

Implementation of virtual communities is strongly influenced by organizational readiness. Many health care organizations lack sufficient internal capacity to support these systems, particularly regarding technological infrastructure, workforce expertise, and coordinated workflows [34,35]. Only a few studies reported collaboration between IT and mental health departments, and there is limited evidence of integrated organizational strategies or defined roles for digital health implementation [35]. Employment of technical professionals and systematic training is often inadequate, suggesting virtual initiatives are frequently treated as add-ons rather than strategic priorities (see Multimedia Appendix 3 for details).

Organizational responsiveness to user feedback and evolving operational challenges is generally limited. Some projects, such as WeCanManage and iaya, demonstrated improvements through iterative system updates [34,35].

### Domain 6: Wider System (External Factors)

Many studies report that privacy and data protection are often overlooked, as virtual communities operate within local legal contexts with limited regulatory oversight (see Multimedia Appendix 3 for details). Social media platforms may offer built-in privacy features, whereas app-based platforms face stricter obligations under health data regulations, such as the GDPR in Europe [30], particularly when handling sensitive patient information.

Cultural and linguistic barriers can reduce user engagement and hinder equitable access, particularly in multicultural or multilingual settings. For example, some platforms offer only English-language interfaces, reflecting limited adaptability to diverse user populations [30].

Technological environment factors, including smartphone penetration, internet access, and the availability of supporting tools such as free activity-tracking apps, are underexplored (see Multimedia Appendix 3 for details). Studies rarely assess whether virtual communities function reliably across different digital infrastructures.

Discrepancies between technical simulations and actual user experiences suggest that current systems may have limited environmental adaptability, which can restrict real-world functionality and scalability [34,35]. At least 2 studies reported strategies to promote community engagement, such as modular learning formats and hybrid models linking online and offline support [35,51].

### Domain 7: Embedding and Adaptation Over Time

#### Long-Term Usage and Evolving User Needs

Needs of patients with cancer evolve throughout the course of the disease, and virtual communities address different priorities at each stage. During diagnosis, patients primarily seek information about disease and treatment options, as observed in platforms such as WeCanManage [35]. In the treatment phase, emotional support, management of side effects, and guidance on lifestyle adjustments become more prominent, which are provided by apps like WalkON [30]. During rehabilitation, the need for sustained psychological assistance and peer interaction continues, with platforms such as Kræftværket offering long-term support and opportunities for experience sharing [31]. In later stages, emotional comfort and end-of-life care are central concerns for patients and their families [33].

Distinct patient populations exhibit specific needs. Adolescents and young adults rely heavily on peer interaction and peer-based support [31], while patients with breast cancer engage in psychological processes including mirroring, monitoring, modeling, belonging, and distancing [37]. User engagement also varies by stage of participation. New users generally focus on information seeking with limited interaction, whereas long-term users tend to adopt active roles, providing guidance and emotional support. Data from platforms such as CSN indicate that 62.5% of registered users disengage after their first login, while a smaller cohort contributes to emotional continuity by welcoming and supporting newcomers [40,43].

#### Technological Updates and Continuous Optimization

Many virtual communities lack mechanisms to accommodate evolving patient needs, limiting their ability to provide stage-specific support across different illness trajectories (see Multimedia Appendix 3 for details). Yao et al reported that most platforms do not deliver interventions tailored to disease stages, highlighting gaps in dynamic and responsive design [43].

Virtual communities often operate separately from formal health care systems, with limited integration between community-based support and formal care infrastructures. Tripathi et al suggested that social media platforms or anonymous forums, such as Reddit, could be integrated into personalized palliative care plans by health care providers [52].

## Discussion

### Principal Findings

This study, guided by the NASSS framework, examined the role of virtual communities in the psychosocial rehabilitation of patients with cancer over the past 5 years. Virtual communities were found to be instrumental in emotional regulation, self-empowerment, and the alleviation of loneliness. Emotional support reduces feelings of isolation, while the sharing of personal experiences contributes to increased confidence and optimism [43,51]. Virtual communities provide informational support, enhancing patients’ sense of belonging and purpose while offering a safe space to express negative emotions and reduce psychological stress [36,44]. Female participants tend to be more active, possibly reflecting patterns of gender socialization [55].

Notably, the predominance of qualitative and mixed methods studies, often employing thematic analysis, semistructured interviews, and emerging approaches such as netnography and text mining, reflects a growing integration of technology-driven methods with traditional qualitative research. This trend highlights an increased emphasis on user experience in the digital context of virtual communities and informs the interpretation of psychosocial outcomes.

The predominance of breast and pan-cancer studies, coupled with the underrepresentation of cancers such as lung, thyroid, or colorectal, highlights imbalances that may restrict generalizability. Similarly, participation patterns show bias toward younger, digitally literate, and female patients, whereas older adults, men, and socioeconomically disadvantaged groups are underrepresented. Psychological comorbidities are also rarely examined. These gaps emphasize the need for more inclusive and representative virtual community designs (Domain 1).

Applying the NASSS framework revealed the dynamic interplay between technological design, policy context, and evolving patient needs. From an implementation science perspective, these findings emphasize the need to coordinate technological and social components to better integrate virtual communities into health care systems. Beyond describing current practices, this study also sheds light on how virtual communities can address personalized needs, deliver sustained support, and promote long-term development. These insights inform strategies to enhance the scalability, adaptability, and sustainability of digital support systems.

### Challenges and Recommendations

#### Adapting to Patients’ Dynamic Needs

Needs of patients with cancer evolve significantly across different stages of their illness trajectory, including diagnosis, treatment, and rehabilitation [56]. In the diagnostic stage, patients primarily seek authoritative medical information and emotional reassurance, while those in rehabilitation are more concerned with long-term psychological support and opportunities to share experiences [31,37]. Evidence indicates that virtual communities often lack dynamic, stage-specific support and mechanisms to help patients transition from passive information seekers to active contributors are rarely described (Domain 7).

To address this issue, a structured “phased needs model” could guide content delivery and interaction design across the cancer care continuum. Real-time interactive features could be prioritized during active treatment, while curated experience repositories may better serve patients in rehabilitation. Artificial intelligence–based systems that analyze user behaviors, such as browsing patterns and content engagement, can help personalize recommendations according to stage-specific concerns and emotional needs [57,58]. This type of modular and responsive design increases the relevance of support, enhances engagement, and promotes continuity of care. Scalability and sustainability depend on coordination among health care providers, platform developers, and health administrators to embed these dynamic features into practice [23].

#### Addressing Disparities in Access and Participation

Rural populations, men, and ethnic minorities remain underrepresented in virtual community research, limiting inclusiveness and generalizability. Studies are concentrated in North America, Europe, Australia, and Asia, reflecting technological and funding disparities. Participation is further shaped by demographic and psychosocial factors: AYA patients rely heavily on peer-based support, while women facing reproductive or identity-related concerns may benefit from tailored community designs. These findings highlight the value of subgroup-specific customization to enhance relevance and equity (Domain 3).

To address these gaps, participatory codesign involving patients, survivors, and health care providers can help ensure that platforms reflect diverse communication styles, cultural norms, and digital limitations [59]. Features such as voice-based or SMS-compatible functions, simplified navigation, and low-bandwidth optimization can expand accessibility for older adults, migrants, low-literacy users, and those in resource-limited rural areas [60,61]. Embedding digital navigators or peer mentors can provide stepwise guidance and ongoing support, which is particularly valuable for male users who may be less likely to seek help proactively [62]. Cultural and linguistic adaptations such as multilingual interfaces, localized educational materials, and culturally tailored survivorship apps have been shown to increase trust and engagement among minority groups [63,64]. Finally, incorporating equity-oriented evaluation metrics, including participation and retention across demographic subgroups, can help monitor inclusivity and guide iterative improvements [65].

#### Integrating Technology with Psychological Support

While anonymity and real-time interaction are common in virtual communities, their effects on psychological well-being remain underexplored. Anonymity reduces expression pressure but may weaken trust and belonging, whereas pseudonymity encourages open discussion but can hinder sustained engagement and deeper relationships. Integrating health care providers as moderators enhances legitimacy and fosters reliable participation [45] (Domains 2 and 4).

Cultural differences influence preferences for anonymity and support mechanisms, reflecting collectivist versus individualist orientations [66,67]. Platforms with single-language interfaces or lacking cultural adaptability risk reducing engagement in multicultural or multilingual populations, highlighting the need for culturally and linguistically responsive design (Domain 6).

To address these challenges, layered anonymity mechanisms can enhance trust while maintaining privacy [68]. New users can engage with minimal identity disclosure and gradually increase visibility as participation deepens. Culturally sensitive features, including group-based support for collectivist cultures, personalized recommendations for individualist cultures, multilingual support, and localized content, may improve psychological connection and inclusivity. Despite this importance, few virtual communities provide systematic cross-cultural adaptation, limiting scalability and engagement (Domains 2 and 6).

#### Addressing Long-Term Impact and User Retention

Longitudinal research on the psychological and behavioral effects of virtual communities is limited, and many platforms face low engagement and retention. The “lurker” phenomenon, where users passively consume content without contributing, hinders community vibrancy, whereas a small group of long-term users provides emotional continuity by welcoming and supporting newcomers [38,40,41,45,48,54]. Standardized metrics such as login frequency, retention rates, and active user proportions are rarely employed, limiting cross-platform comparisons and evaluation of effectiveness (Domain 7).

Although initial engagement is often driven by novelty or immediate needs, the lack of sustained incentives can reduce long-term activity [69]. To address this, platforms could implement reward systems (eg, contribution leaderboards or shareable badges), support gradual role transitions from information-seeking to mentorship, provide regular content updates, and conduct user surveys to foster belonging and community relevance. Dynamic content strategies that integrate continuous user feedback loops can reinforce perceived value and emotional connection. Establishing longitudinal frameworks to track psychological states, quality of life, and engagement patterns enables continuous optimization of community features. Artificial intelligence–driven behavioral analysis can further personalize content recommendations and interaction prompts, supporting long-term stickiness and tailored psychosocial support [43,58,69,70] (Domain 7).

#### Policy and Organizational Support

Inadequate data privacy measures and regulatory frameworks hinder the scalability and sustainability of virtual communities. Strict adherence to regulations such as the GDPR and the Health Insurance Portability and Accountability Act is essential, yet technological advancements often outpace policy adaptations, creating a regulatory mismatch [71]. Limited organizational capacity and insufficient cross-departmental collaboration further constrain implementation. Platforms embedded within institutional learning systems, such as WeCanManage and iaya, demonstrate greater adaptability and sustainability by incorporating user feedback and cross-functional governance (Domains 5 and 6).

Technological solutions, including blockchain and differential privacy models, can enhance data security while maintaining transparency and enabling research. Blockchain ensures data integrity, whereas differential privacy balances confidentiality with data analysis [68,72]. Proper implementation of these tools can build user trust and support evidence-based platform development.

Collaborations between health care institutions and technology companies can enhance credibility and professionalism. Such partnerships bridge clinical standards and technological innovation, enabling the cocreation of responsive and ethically sound support environments. Establishing cross-departmental task forces facilitates resource sharing and ensures responsiveness to user needs and evolving policies.

#### Ethical Considerations for Emotional Exposure in Virtual Communities

In addition to privacy and governance, virtual communities raise ethical concerns related to emotional safety. Emotional contagion, in which exposure to others’ distress increases users’ own anxiety, may disproportionately affect newly diagnosed or emotionally vulnerable individuals [73-75]. Open platforms can also expose users to unverified treatments, exaggerated negative experiences, and algorithm-driven content repetition, which may amplify distress, reinforce negative emotions, narrow perspectives, and influence treatment decisions (Domains 2 and 4).

To address these risks, platforms should implement emotion-sensitive governance strategies. These strategies include flagging harmful content, promoting balanced emotional narratives, and using content curation tools that prioritize emotional safety. Establishing a supportive and trustworthy environment is essential for maintaining user engagement, psychological well-being, and long-term participation.

### Future Direction

Virtual communities play a vital role in patient empowerment, health management, psychological support, and health policy development. By providing personalized health information, emotional support, and interactive engagement, these platforms significantly enhance patients’ sense of agency and capacity for self-management, particularly in chronic illnesses such as cancer [76-78].

Future research should include underrepresented populations and regions, as current evidence is concentrated in North America and skewed toward younger, female, and digitally literate patients. Designing culturally responsive and demographically inclusive virtual communities can improve representativeness and health equity (Domains 1, 3, and 6). A key research focus is understanding how virtual communities promote empowerment and influence adherence and long-term outcomes. Integration with clinical workflows and interoperability with health care systems should be prioritized to maximize longitudinal benefits and scalability (Domain 7). It is also essential to design support programs that address the needs of specific populations, including LGBTQ+individuals, people with low income, and ethnic minority groups, to promote equity and inclusivity [79,80].

In mental health, virtual communities offer scalable opportunities for psychological support. Using natural language processing and machine learning, platforms can identify users’ emotional states in real-time and provide targeted interventions, especially during public health crises like COVID-19 [78,81]. Simultaneously, the risk of misinformation remains a critical challenge. Future development should integrate health information verification systems, credibility scoring mechanisms, and strategies to enhance users’ health literacy, enabling them to identify reliable information [78,82].

Technological innovation and personalized, data-driven support are important directions for advancement. Community-generated data can support intelligent recommendation systems and digital tools that anticipate user needs, improving health management [13,83]. Large-scale data analysis may also reveal unmet patient needs and inform health policy refinement [84]. These developments must be accompanied by strong data privacy protections and ethical safeguards, including anonymization, encryption, and standardized frameworks for secure data sharing [78]. Properly implemented, community-generated data can inform intelligent recommendations and policy-relevant insights while maintaining sustainable, trustworthy, and inclusive digital support systems (Domains 2, 5, and 6).

### Study Limitations

This review has several limitations related to the methodological heterogeneity of the included studies. The sample comprised diverse research designs, including qualitative, quantitative, and mixed methods studies, each contributing different types of evidence. Qualitative studies offered rich, in-depth insights into user satisfaction, emotional support, and perceived implementation barriers but generally involved small, self-selected samples and relied on self-reported data, which may introduce selection and reporting biases, limiting generalizability.

Quantitative studies employed standardized measures to assess outcomes such as psychological well-being and user engagement, providing broader representativeness but often lacking the contextual depth offered by qualitative data. Mixed methods studies facilitated integration of these perspectives, although variations in study quality and inconsistent reporting constrained comprehensive synthesis. The heterogeneity in study designs, data collection methods, and outcome measures posed challenges for interpretation, as qualitative research emphasizes subjective experiences while quantitative data focus on measurable outcomes. Consequently, some nuanced barriers or facilitators, including organizational capacity or psychosocial factors, may be underrepresented in quantitative analyses but detailed in qualitative narratives.

To address this, we applied a structured, framework-guided approach based on NASSS domains to systematically integrate diverse data. While this enabled thematic convergence, the lack of uniform outcome measures and inconsistent reporting limited direct comparisons or meta-analysis. A key limitation is the predominance of descriptive data on implementation features such as emotional experience, user trust, and perceived usability. Although crucial to adoption and sustainability, these factors remain undertheorized and seldom operationalized quantitatively. The scarcity of quantitative engagement metrics, including retention and active participation rates, limits rigorous assessment of long-term effectiveness and adherence.

Additionally, our search was limited to English-language studies published within the last 5 years, potentially excluding relevant work in other languages or earlier periods. Finally, the predominantly cross-sectional nature of the included studies restricts insights into the long-term impact and sustainability of virtual communities.

### Conclusions

This study uses the NASSS framework to explore the application, challenges, and optimization of virtual communities in the psychosocial rehabilitation of patients with cancer. The findings highlight the important role of virtual communities in providing psychological support, facilitating information sharing, and reducing loneliness, leading to improvements in emotional regulation and self-empowerment. Nonetheless, significant challenges persist, including effectively meeting patients’ evolving needs, integrating technological tools with psychological support, and securing long-term efficacy alongside supportive policy frameworks.

To overcome these challenges, it is essential to implement dynamic closed-loop mechanisms that enable continuous adaptation, promote ongoing technological innovation, develop diversified and culturally sensitive designs, and ensure robust policy support. Future research should prioritize longitudinal studies to capture changes over time, conduct cross-cultural comparisons to address diversity, and explore the integration of emerging technologies to improve the sustainability and effectiveness of virtual communities in cancer rehabilitation. With sustained research efforts and iterative enhancements, virtual communities hold great promise to substantially improve the quality of life and rehabilitation outcomes for patients with cancer.

## Supplementary material

10.2196/73093 Multimedia Appendix 1 Search strategy.

10.2196/73093 Multimedia Appendix 2 Literature Exclusion Reasons Summary Form.

10.2196/73093 Multimedia Appendix 3 Data extraction scale.

10.2196/73093 Multimedia Appendix 4 Data Extraction Matrix with NASSS-Based Domain Mapping and Interpretation.

10.2196/73093 Multimedia Appendix 5 MMAT assessment results.

10.2196/73093 Checklist 1 PRISMA-ScR Checklist.

## Abbreviations

AYA: adolescent and young adult
CCOC: Cancer Council Online Community
CSN: Cancer Survivor Network
GDPR: General Data Protection Regulation
MeSH: Medical Subject Headings
MMAT: Mixed Methods Appraisal Tool
NASSS: Non-adoption, Abandonment, Scale-up, Spread, and Sustainability
PRISMA-ScR: Preferred Reporting Items for Systematic Reviews and Meta-Analyses extension for Scoping Reviews
