# Supplementary material for: Mapping Characteristics, Applications, and Implementation Challenges of Virtual Communities in Cancer Care: NASSS Framework-Informed Scoping Review
Source: J Med Internet Res. 2025 Oct 22;27:e73093. doi: 10.2196/73093 (PMC12543217; doi:10.2196/73093)
Supplement: Multimedia Appendix 1 [file jmir-v27-e73093-s001.pdf]

Multimedia Appendix 1. Search strategy

1. Database and keyword:

（1） Database: PubMed, EMBASE, SCOPUS, Web of Science (core collection), EBSCOhost, PsycINFO

（2） Keywords and Medical Subject Headings (MeSH) terms:

1) virtual community, online community
2) cancer, carcinoma, neoplasm, cancer survivors, cancer patients, oncology patient
3) psychology, psychosomatic, somatic

2. Boolean logic combination:

（1） PubMed: ((Virtual community[tiab]) OR (Online community[tiab]) OR (APP[tiab] AND community[tiab])) AND ((Cancer[MeSH Major Topic]) OR (Carcinoma[MeSH Major Topic]) OR (Neoplasm[Mesh]) OR (Cancer Survivor[Mesh]) OR (Cancer Patient[MeSH Major Topic]) OR (Oncology Patient[MeSH Major Topic]))

（2） EMBASE: ('virtual community' OR 'online community') AND ('cancer'/exp OR 'cancer' OR 'carcinoma'/exp OR 'carcinoma' OR 'neoplasm'/exp OR 'neoplasm' OR 'cancer survivor'/exp OR 'cancer survivor')

（3） SCOPUS: (TITLE-ABS-KEY (virtual-community OR online-community) AND TITLE-ABS-KEY (cancer OR neoplasm OR

carcinoma OR cancer-survivor OR cancer-patient OR oncology-patient)) AND PUBYEAR > 2019 AND PUBYEAR < 2025 AND (LIMIT-TO (DOCTYPE , "ar") OR LIMIT-TO (DOCTYPE , "re")) AND (LIMIT-TO (LANGUAGE , "English"))

（4） Web of Science (Core Collection): TS=("Virtual community" OR "Online community") AND TS=("Cancer" OR "Carcinoma" OR "Neoplasms" OR "Cancer Survivors" OR "Cancer Patient" OR "Oncology Patient")

（5） EBSCO host: SU (virtual community OR online community OR virtual communities) AND SU (cancer patient OR oncology patient OR cancer survivor)

（6） PsycINFO: SU (virtual community OR online community OR support-group) AND SU (cancer patient OR oncology patient OR cancer survivor)
