# Supplementary material for: Mapping Characteristics, Applications, and Implementation Challenges of Virtual Communities in Cancer Care: NASSS Framework-Informed Scoping Review
Source: J Med Internet Res. 2025 Oct 22;27:e73093. doi: 10.2196/73093 (PMC12543217; doi:10.2196/73093)
Supplement: Multimedia Appendix 4 [file jmir-v27-e73093-s004.pdf]

Data Collection Extraction Entries: Based on the NASSS Framework

1. Basic Information

（1）Title:

（2）Publication Year:

（3）Source Country:

（4）DOI URL:

2. Research Design Information

（1）Research Type:

（2）Abstract:

（3）Intervention Group (Or Total Sample):

（4）Control Group:

（5）Intervention Time:

3. Characteristics of Virtual Community

（1）Name of virtual community (application):

（2）Type of virtual community:

（3）Virtual Community Introduction, Features:

（4）Type of social support:

（5）Emotional support approach:

（6）Negative impacts due to virtual communities:

4. <u>Domain 1：Condition</u>

（1）Type of Cancer:

（2）Disease Stage:

（3）Emotional Baseline Conditions:

（4）Patient's Age Stage:

（5）Co-Morbidities:

（6）Gender Composition:

（7）Race, Ethnicity:

（8）Socio-Economic Status:

（9）Impact Of The COVID-19 Pandemic:

5. <u>Domain 2：Technology</u>

（1）Participating devices (cell phones, tablets, computers):

（2）User type:

（3）Interaction methods (interactive boards, message boards, real-time messaging, calls):

（4）Anonymized or not (reason):

（5）Degree of automation of interventions:

（6）supervision and guidance:

（7）Personalized design (content, form):

（8）AI-driven or not:

（9）Patient data collection methods:

（10）Patient data management (privacy protection):

（11）Technical characteristics (complexity, user-friendliness of the interface, variety of functions, cross-platform compatibility):

6. <u>Domain 3：Value Proposition</u>

（1）Demand side value (patient demand for virtual community psychosomatic rehabilitation):

（2）Supply-side value and sustainability (maintenance costs, operating model, development potential):

7. <u>Domain 4：Adopters</u>

（1）Patient needs and expectations

1) Prioritized needs and willingness to use among different patient groups:

2) Patient experience and satisfaction (interface design, ease of use, personalization, barriers):

（2）The Role of Healthcare Providers

1) Involvement of medical providers (technical support, counseling, treatment advice):

2) Healthcare provider attitudes toward technology, acceptance, barriers difficulties:

（3）The role of technology developers

1) Engagement process for technology developers:

2) Views of technology developers:

8. <u>Domain 5：Organization</u>

（1）Team composition:

（2）Availability of relevant training:

（3）Cross-sectoral collaborative working models:

（4）Flexibility to respond to operational issues and user feedback:

（5）Proactive continuous improvement at the organizational level:

9. <u>Domain 6：Wider System</u>

（1）Privacy and Data Protection Legislation:

（2）Data regulation and compliance:

（3）Influence of the socio-cultural context:

（4）Technology infrastructure (Internet penetration, network speed, device availability):

（5）Technical compatibility and interoperability:

（6）Building and utilizing local social support networks:

（7）Designs to enhance community participation:

10. <u>Domain 7：Embedding and Adaptation Over Time</u>

（1）Dynamics of needs at different stages of illnesses:

（2）Adaptability to dynamic changes in demand:

（3）Ability to continuously iterate on technology:

（4）Integration with other healthcare management systems:

Supplementary Notes

1. Operationalizing NASSS Domains in Data Extraction

To improve methodological transparency and reproducibility, this section details how the seven domains of the NASSS framework (Non-adoption, Abandonment, Scale-up, Spread, and Sustainability) were applied to guide data extraction. The aim of this mapping was to systematically organize implementation-related content from a diverse body of literature into a coherent structure that enables both thematic comparison and conceptual synthesis.

Each NASSS domain was operationalized into specific data extraction fields within the matrix (see above). These fields were developed by the research team through a deductive interpretation of the original NASSS model and tailored to the specific context of virtual communities for cancer care. In this appendix, we outline the conceptual scope of each domain, illustrate how it is commonly reflected in the literature, and clarify its corresponding indicators in the data extraction process.

Domain 1: Condition

Definition:

This domain refers to the complexity of the health condition targeted by the intervention, encompassing its clinical presentation, psychosocial dimensions, and contextual factors. These characteristics influence how individuals engage with virtual community platforms.

Application in review:

In the context of this study, the domain captures how various features of cancer shape patient engagement in virtual communities. Relevant aspects include the type of cancer (such as breast, colorectal, or rare forms), disease stage (such as terminal or post-treatment survivorship), physical symptoms (such as pain and fatigue), and psychological conditions (such as anxiety and loneliness). It also accounts for comorbidities and other demographic or illness-related variables that influence patient needs and participation.

Data extraction fields aligned with this domain:

- Cancer type: Identifying the type of cancer (e.g., breast, colorectal, or rare cancers).
- Treatment stage: Considering the stage of the disease (e.g., terminal, active treatment, or post-treatment survivorship).

- Emotional vulnerabilities: Addressing psychological factors such as anxiety, fear of recurrence, or loneliness.
- Demographic characteristics relevant to participation: Identifying key demographic or illness-related factors (e.g., age, gender, or comorbidities) that may influence patient engagement and participation.

Example entries:

- "Participants included women with stage IV metastatic breast cancer."
- "Many users reported post-treatment fear of recurrence."

Domain 2: Technology

Definition:

This domain focuses on the technical design, usability, and system compatibility of the virtual community platform.

Application in review:

This domain focuses on how technological characteristics of virtual communities influence user experience and engagement. It includes the type of platform used (such as mobile apps, online forums, or social media groups), interface design, ease of use, and the presence of functional modules such as chat tools, discussion sections, or peer matching. It also covers security-related aspects, including privacy settings and data protection mechanisms, as well as technical issues that may affect platform stability or access, such as login difficulties or functional errors.

Data extraction fields aligned with this domain:

- Platform format (e.g., app, forum, social media group): Identify the type of platform used to host the virtual community.
- Feature design (e.g., chat, matching, reminders): Extract information about specific functionalities, such as whether the platform includes chat tools, reminder systems, or matching features
- Usability and accessibility: Evaluate how easy it is for users to navigate the platform and whether it is accessible across various devices or systems.
- Privacy policy or data encryption: Assess the platform's data protection mechanisms, including whether it uses encryption and if it has a clear privacy policy.

- Technical reliability: Consider the technical stability of the platform, including potential issues like login difficulties, slow load times, or system errors.

Example entries:

- “The app included symptom-tracking and mood journaling functions, providing users with interactive features to track their mental health.”
- “Users expressed frustration with slow-loading discussion boards, which affected their participation in the community.”

Domain 3: Value Proposition

Definition:

This domain refers to the perceived benefits and value of the virtual community platform for its users, including emotional, functional, informational, and motivational aspects.

Application in review:

This domain captures both subjective perceptions and reported outcomes related to users’ experiences with virtual communities. It includes descriptions of emotional support, a sense of empowerment, the usefulness of shared information, and the perceived quality of peer interaction. It also reflects user-reported limitations, such as unmet expectations, lack of personalization, or concerns about misinformation and redundancy.

Data extraction fields aligned with this domain:

- Perceived usefulness: How users perceive the utility of the platform for meeting their needs (e.g., emotional support, functional assistance, or informational value).
- Reported emotional or functional outcomes: User experiences such as improved emotional well-being or functional support (e.g., coping mechanisms, empowerment).
- Satisfaction with content or interaction: Users' satisfaction with the quality and relevance of content and interactions within the community.
- Value gaps (unmet needs): Limitations reported by users, such as unmet expectations, lack of personalization, or concerns about misinformation or redundancy.

Example entries:

- “Helped reduce my fear of being alone.”

- “Too generic—didn’t offer anything I couldn’t get from Google.”

Domain 4: Adopters

Definition:

This domain concerns the characteristics, behaviors, and attitudes of individuals who engage with the virtual community, including patients, caregivers, and healthcare professionals.

Application in review:

This domain captures how users interact with virtual communities and the factors influencing their level of engagement. It includes different usage patterns, such as active participation or passive observation (often referred to as “lurking”), as well as digital literacy, motivations for joining or withdrawing, and emotional responses to participation. It also considers the role of professionals, such as clinicians or moderators, and how demographic or cultural differences shape user behavior.

Data extraction fields aligned with this domain:

- Usage type (active/passive): The nature of participation, such as active engagement (posting, commenting) or passive observation (often referred to as “lurking”).
- Peer interaction modes: How users engage with others in the community, such as through direct communication or indirect observation.
- Motivation for joining: Reasons for participation, including emotional support, information seeking, or community belonging.
- Professional involvement: The role of healthcare professionals, such as clinicians or moderators, in guiding or facilitating interactions.
- Emotional responses to participation: Users’ emotional experiences, including feelings of empowerment, connection, or distress arising from engagement.

Example entries:

- “Users described ‘lurking’ as a safe way to learn from others without emotional exposure.”
- “A cancer nurse specialist moderated the group and intervened when misinformation appeared.”

Domain 5: Organization (Internal Factors)

Definition:

This domain refers to the internal organizational structure that supports the operation of the virtual community. It includes aspects such as content moderation, institutional involvement, allocation of resources, and the extent to which the platform is integrated into existing models of healthcare delivery.

Application in review:

This domain examines the nature of the organization responsible for managing the virtual community, which may include hospitals, nonprofit organizations, or informal patient-led initiatives. It also addresses how content is moderated, how quality assurance is maintained, and whether the platform operates independently or is linked to formal clinical services or support systems.

Data extraction fields aligned with this domain:

- Platform governance (hospital, non-profit, informal): Identifies the type of organization managing the platform (e.g., hospital, non-profit, or informal patient-led).
- Moderator role (peer-led, clinician-led): Describes who moderates the community (e.g., peer support or clinician-led).
- Resource allocation: Refers to the availability and distribution of resources for maintaining the platform.
- Quality control protocols: Indicates the processes for ensuring content quality and accuracy.
- Integration with offline services: Assesses whether the platform is linked to formal healthcare services or provides offline support.

Example entries:

- "Run by a cancer center with trained nurses monitoring discussions."
- "The platform partnered with a nonprofit organization to provide weekly content review and emotional support sessions."

Domain 6: Wider System (External Factors)

Definition:

This domain refers to the broader external environment in which the virtual community is situated, including sociocultural norms, legal and regulatory frameworks, technological infrastructure, and health system characteristics.

Application in review:

This domain captures factors beyond the platform or user level that influence the adoption and use of virtual communities. These include disparities in digital literacy, the presence of national or regional data protection regulations, cultural attitudes toward illness and emotional expression, limitations in internet or device access, and language-related barriers. It also considers broader health system constraints that may limit the integration or uptake of digital support platforms.

Data extraction fields aligned with this domain:

- Cultural and linguistic accessibility: Whether the platform accommodates different languages and cultural norms.
- Internet or device access: Accessibility of the platform given varying levels of internet connectivity or device availability.
- Privacy regulations and legal concerns: Considerations around compliance with data protection laws and privacy regulations.
- Regional health system constraints: Barriers in the local health system that may limit the platform's adoption or integration.

Example entries:

- "Patients in rural areas struggled to maintain stable connections."
- "In some cultures, users were hesitant to discuss psychological needs."

Domain 7: Embedding and Adaptation Over Time

Definition:

This domain addresses how virtual communities evolve over time in response to user needs, clinical developments, and external environmental changes. It encompasses considerations of sustainability, platform modification, and long-term relevance.

Application in review:

This domain focuses on the extent to which platforms are maintained, adapted, or updated based on user feedback and changing circumstances. It includes observations on how content and design evolve, whether mechanisms are in place to support continued user engagement, and how platforms remain relevant as patients transition between different phases of their cancer journey. The domain also considers behavioral patterns such as disengagement, return, or re-engagement at different stages.

Data extraction fields aligned with this domain:

- Platform evolution or redesign: Whether the platform undergoes changes or improvements over time.
- Feedback integration: How user feedback is incorporated into the platform's design and features.
- Strategies to maintain engagement: Mechanisms in place to keep users involved over the long term, such as content updates or personalized experiences.
- Personalization mechanisms: Features that allow for the platform to adapt to individual user needs.
- Dropout and return behaviors: Patterns of disengagement and re-engagement at various stages, especially when patients transition between phases of their cancer journey.

Example entries:

- "After feedback, the platform added a relapse discussion section."
- "Users disengaged after remission and rejoined during recurrence."

2. General Study Characteristics (Outside NASSS Framework)

In addition to NASSS-guided fields, we extracted general information for each study to provide contextual background for synthesis. These include:

- Study characteristics: Author, publication year, country, study design, data collection method, sample size (if applicable).
- Participant characteristics: Cancer type, treatment status, demographic information (e.g., age, gender, minority group status).
- Platform characteristics: Platform name or type, developer or host, openness (closed/private vs. open), technological channel (web, mobile app, social media).

3. Instructions for Filling Data Extraction Matrix

This section provides comprehensive guidance on how to systematically extract and record data from studies into the data extraction matrix. These steps are designed to ensure consistency and accuracy while capturing all relevant features of the studies. Please follow the instructions below:

1. **Feature Alignment**

   Begin by evaluating feature alignment. This step involves assessing how well the core attributes of the virtual communities align with the intended goals and needs of the users.

- **Why is this important**: Understanding whether a platform's design supports its stated objectives is crucial for evaluating its effectiveness. If a study is focused on emotional support, for instance, the platform should include tools and features that enable communication, peer support, and community building.
- **How to assess**: Look for features such as user interaction tools (e.g., chat, video calls), social networking capabilities, information-sharing functionalities, or any mechanisms that promote user engagement and collaboration. These features should align with the intended purpose of the community, such as providing support, offering information, or fostering a sense of belonging.
- **Example**: If the study highlights the use of a mobile app for cancer patients, assess whether the app includes features such as personalized support groups, reminders, or access to medical resources. These would demonstrate alignment with the platform's goals.

2. **Extract Relevant Feature Entries**

After evaluating feature alignment, proceed to extract relevant feature entries from the study. This process involves identifying and summarizing the key elements of the virtual community that are pertinent to your analysis.

- **Technological Features**: These include the type of platform (e.g., web-based, mobile application, or social media), as well as specific technological tools that facilitate user interaction, engagement, or content sharing. You should also note any unique technical aspects, such as integration with health data, accessibility features (e.g., text-to-speech, language options), or any specialized tools designed for specific user groups (e.g., patients with mobility challenges).
- **User Engagement Strategies**: Identify how the platform engages users. This might include automated messages, feedback loops, motivational tools, gamification elements, or peer support systems. Record how these strategies are designed to keep users active, involved, and engaged with the platform over time.
- **Content Structure**: Examine how the platform organizes its content. Does it include discussion boards, educational videos, or articles? Are the resources categorized to make it

easier for users to find relevant information? Ensure you capture how the content is structured to facilitate ease of use and engagement.

- **Example**: If the study mentions a web-based community platform offering daily health tips and articles, make sure to include these details along with any interactive elements that encourage users to participate in discussions or share personal stories.

3. **Handling No Relevant Content**

In some cases, the study may not provide information on certain features or domains. When this happens, you should mark the corresponding cell in the matrix as "No relevant content".

- **Why this is important**: Clearly marking cells where data is missing helps maintain transparency and ensures that gaps in the data are properly documented. This is especially important when synthesizing multiple studies, as it prevents the inclusion of incomplete or misleading data.
- **How to apply**: If a study focuses on clinical outcomes without touching on platform characteristics, user engagement, or technological features, you would mark those respective domains as "No relevant content".
- **Example**: If a study discusses the effectiveness of a digital health intervention but provides no information about user engagement features, mark the corresponding User Engagement domain as "No relevant content".

4. **Marking Inapplicable Features or Domains**

Some domains or features may not be conceptually applicable to certain studies. In such cases, mark these sections as "Not applicable".

- **Why this is important**: Not every domain or feature is relevant to all studies. For example, studies involving informal peer-run forums may lack formal organizational structures, making Domain 5: Organization irrelevant.
- **How to apply**: Assess the nature of the platform and the study to determine whether certain domains are not relevant. If the platform is entirely based on user-generated content with no formal oversight, mark organizational domains as "Not applicable".
- **Example**: If the study is about a peer-run forum with no leadership or formal organization, mark the Organization domain as "Not applicable".

5. **Supplementing Information When Needed**

   Occasionally, the original study may not provide enough information on a specific feature or domain. In these cases, supplement the information by conducting web searches for recent data on the referenced virtual communities.

   - **Why this is important**: Many virtual communities evolve over time, adding new features, changing content, or altering user engagement strategies. Supplementing the original study with current data ensures that the data extraction matrix is as accurate and up-to-date as possible.
   - **How to apply**: Perform a web search for the platform or community discussed in the study. Check the platform's website or any other relevant online resources to gather additional details. If new features or updates are found, record these as supplements to the original study.
   - **Example**: If the study mentions a platform from 2015, but recent changes (e.g., the addition of real-time chat or new health resources) have occurred, include these updates in the matrix, noting the source of the updated information.

6. **Dealing with Platform Updates**

   Platforms often undergo updates after a study has been published. When this happens, always use the original study description as the primary reference. However, note any significant changes or updates that may have occurred after the study was published.

   - **Why this is important**: This helps provide a complete picture of how the platform has evolved over time and ensures that the most relevant and current information is included in your analysis.
   - **How to apply**: Check for recent updates to the platform. If new features or changes have been introduced (such as a redesign or the addition of new engagement strategies), mention these in the matrix along with the original study description.
   - **Example**: If the study refers to a platform released in 2018, and the platform has since added a mobile app and integrated virtual consultations in 2022, make sure to note these changes while still referencing the 2018 description for context.

7. **Mapping Domain Coverage**

   Finally, ensure that you map domain coverage systematically for each study. For every NASSS domain, assess whether it has been addressed in the study.

   - **If a domain is addressed**: Extract and summarize the relevant findings in the matrix.

- **If no relevant content is provided**: Mark the cell as "No relevant content". This indicates that the study did not address the domain, ensuring transparency and clarity.
- **If a domain is conceptually irrelevant**: Mark the domain as "Not applicable" if the study does not pertain to it (e.g., if the study does not involve organizational factors or platform engagement).
- **Example**: If a study on a mobile health app does not discuss user engagement or emotional support, mark those domains as "No relevant content".
